# Anatomical considerations for nerve transfer in axillary nerve injury

**DOI:** 10.1038/s41598-024-51923-w

**Published:** 2024-01-13

**Authors:** Soo-Jung Kim, Jong-Ho Bang, Hee-Jun Yang, Seong-Hwan Moon, Yun-Rak Choi, Hye-Yeon Lee

**Affiliations:** 1 Department of Anatomy, Yonsei University College of Medicine, 50-1 Yonsei-ro, Seodaemun-gu, Seoul, 03722 Republic of Korea; 2 Department of Medicine, The Graduate School Yonsei University, Seoul, Republic of Korea; 3 Surgical Anatomy Education Center, Yonsei University College of Medicine, Seoul, Republic of Korea; 4 The Youth Clinic, Bucheon-si, Gyeonggi-do Republic of Korea; 5 Department of Orthopaedic Surgery, Yonsei University College of Medicine, Seoul, Republic of Korea

**Keywords:** Anatomy, Musculoskeletal system

## Abstract

This study investigated the anatomical details of the axillary and radial nerves in 50 upper limbs from 29 adult formalin-embalmed cadavers, and ten fresh upper limbs. The focus was on understanding the course, division, and ramifications of these nerves to improve treatment of shoulder dysfunction caused by axillary nerve damage. The axillary nerve divided anteriorly and posteriorly before passing the quadrangular space in all specimens, with specific distances to the first ramifications. It was found that the deltoid muscle's clavicular and acromial parts were always innervated by the anterior division of the axillary nerve, whereas the spinous part was variably innervated. The longest and thickest branches of the radial nerve to the triceps muscles were identified, with no statistically significant differences in fiber numbers among triceps branches. The study concludes that nerve transfer to the anterior division of the axillary nerve can restore the deltoid muscle in about 86% of shoulders, and the teres minor muscle can be restored by nerve transfer to the posterior division. The medial head branch and long head branch of radial nerve were identified as the best donor options.

## Introduction

The axillary nerve is a branch of the posterior cord of the brachial plexus and contains nerve components from the fifth and sixth cervical spinal segments^1^. It passes through the posterior wall of the axilla via the quadrangular space with the posterior circumflex humeral vessels^2^. The axillary nerve can be injured by blunt trauma, traction injury, penetrating trauma, and nerve compression in the quadrangular space^3^. The most common cause of axillary nerve injury is trauma during orthopedic surgery such as shoulder arthroscopy, thermal shrinkage of the shoulder capsule, and plate fixation on the proximal humerus^4^.

Axillary nerve injury can result in partial or total inactivation of the deltoid and teres minor muscles^4,5^. Because damage to the axillary nerve can result in shoulder instability or dysfunction, it is important to protect the axillary nerve and its branches and to restore the damaged nerves to maintain the functions of shoulder.

Nerve transfer is one of the therapeutic options for functional restoration of denervated muscles^5,6^. To restore the deltoid muscle, nerve transfer using triceps motor branches of the radial nerve to the axillary nerve are widely used^7–10^. The most appropriate choice of branch of the radial nerve to use as the donor and which branch of the axillary nerve to use as the recipient can improve the results of nerve transfer.

The purpose of this study was to understand the anatomy of axillary and radial nerves to prevent nerve damage and restore muscle action. Additionally, this study aimed to identify potential donor branches of the radial nerve that are good matches for potential recipient branches of the axillary nerve in nerve transfer.

## Results

### Branching patterns of the axillary nerve

The clavicular and acromial parts of the deltoid muscle were constantly innervated by the anterior division of the axillary nerve. The teres minor muscle was constantly innervated by the posterior division of the axillary nerve. Variations were observed in the innervation of the spinous part of the deltoid muscle, which was innervated by the posterior division, anterior division, or both.

**Figure 1:**
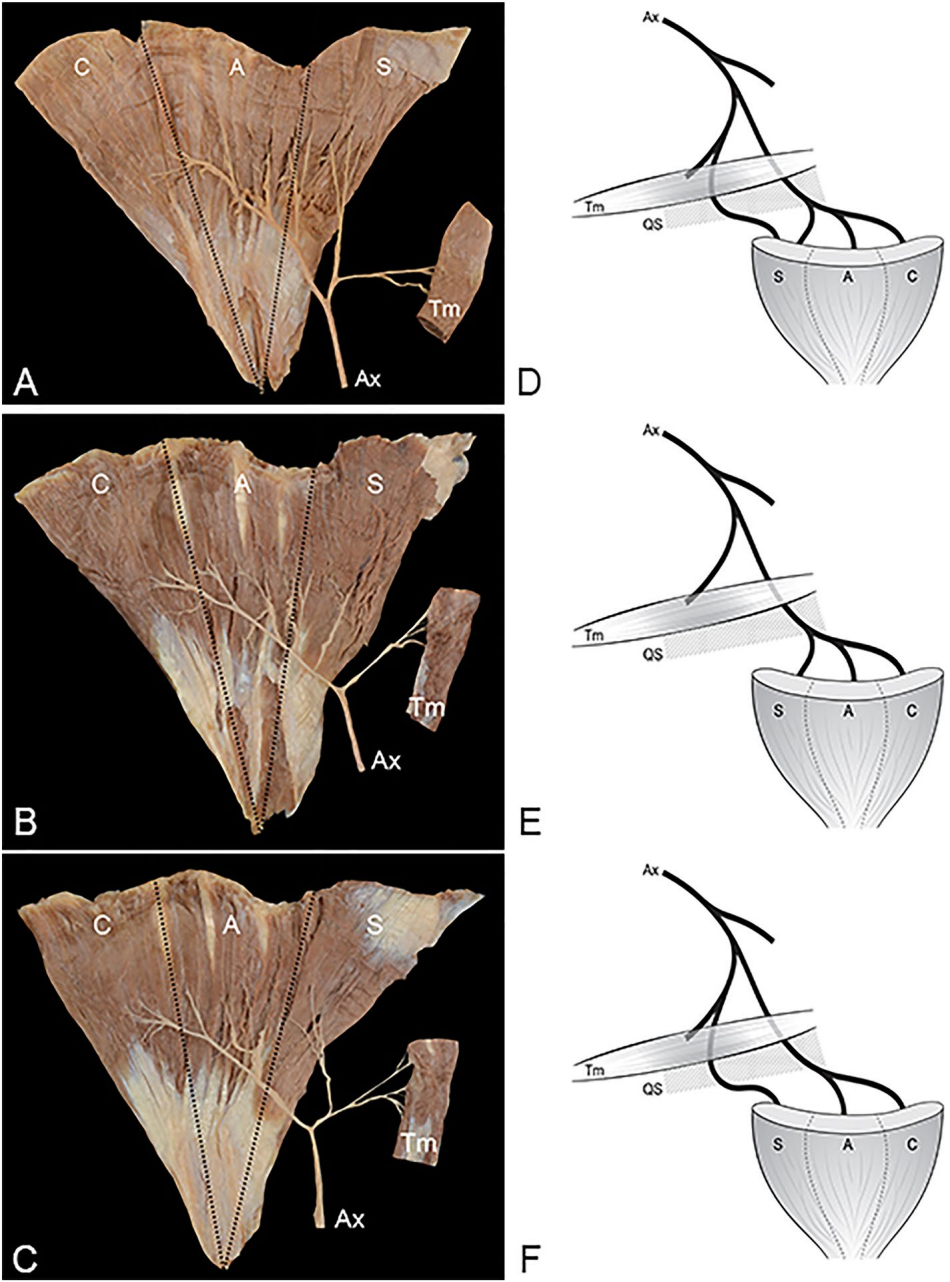
The three types of branching patterns of the axillary nerve to the deltoid and teres minor muscles. (**A,D**) Mixed type: the anterior division innervates all parts of the deltoid and the posterior division innervates both the spinous part of the deltoid and the teres minor; (**B,E**) anterior dominant type: the anterior division innervates all parts of the deltoid, and the posterior division innervates the teres minor; (**C,F**) posterior dominant type: the anterior division innervates the clavicular and acromial parts of the deltoid, while the posterior division innerves the spinous part of the deltoid and the teres minor; *C* clavicular part of the deltoid, *A* acromial part of the deltoid, *S* spinous part of the deltoid, *Tm* teres minor muscle, *QS* quadrangular space.

According to the origin of the nerve branches to the spinous part of the deltoid muscle, variations in the distribution of the axillary nerve to the deltoid and teres minor muscles were grouped into three types (Fig. 1A–F). In 48.0% of upper limbs, the axillary nerve was “mixed type,” as the anterior division of the axillary nerve innervated all parts of deltoid muscle and the posterior division innervated the spinous part of the deltoid muscle as well as the teres minor muscle (Fig. 1A, D). In these upper limbs, the spinous part of the deltoid muscle was innervated by both the anterior and posterior divisions. In 38.0%, the axillary nerve was “anterior dominant type,” as the anterior division innervated all three parts of the deltoid muscle and the posterior division innervated the teres minor muscle only (Fig. 1B, E). In these upper limbs, the spinous part of the deltoid muscle was innervated by only the anterior division. In 14.0%, the axillary nerve was “posterior dominant type” with the anterior division innervating the clavicular and acromial parts of the deltoid muscle, while the posterior division innervated the teres minor and spinous part of the deltoid muscle (Fig. 1C, F).

### Size of the axillary nerve

**Figure 2:**
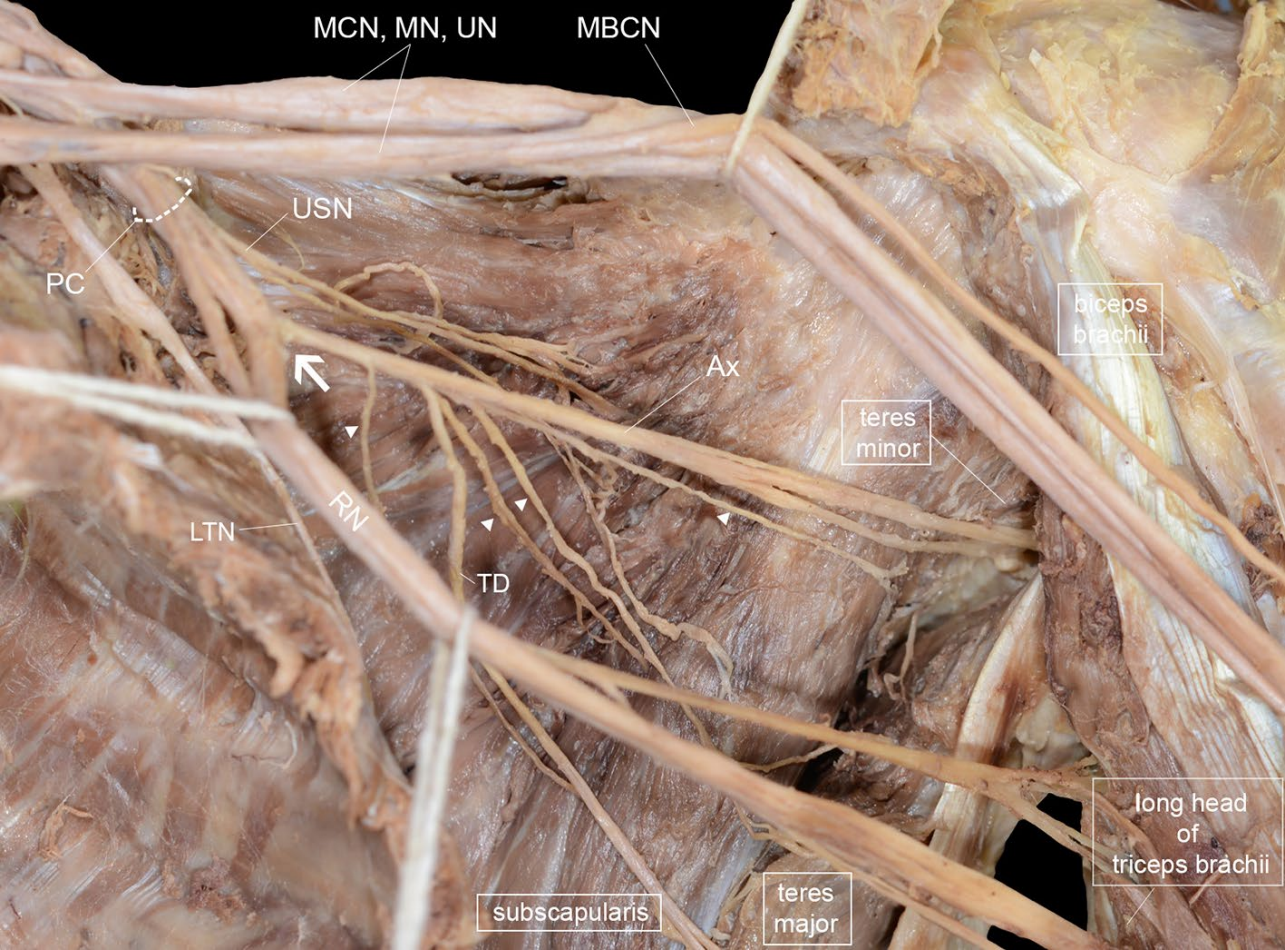
Anterior view of the branches of the axillary nerve (Ax) to the subscapularis and teres minor muscles. The posterior cord of the brachial plexus bifurcates to the radial and axillary nerves (arrow). The axillary nerve branches into lower subscapular nerves (arrowhead) and thoracodorsal nerves. The anterior cords of the brachial plexus and radial nerve are reflected for observation. *MCN* musculocutaneous nerve, *MN* median nerve, *UN* ulnar nerve, *PC* posterior cord, *USN* upper subscapular nerve, *MBCN* medial brachial cutaneous nerve, *TD* thoracodorsal nerve, *LTN* long thoracic nerve, *RN* radial nerve, *Ax* axillary nerve, *SS* subscapularis muscle, *TM* teres major muscle, *Tm* teres minor muscle, *LoH* long head of the triceps brachii muscle.

The axillary nerve bifurcated into anterior and posterior divisions before passing through the quadrangular space in every upper extremity (Fig. 2). The length of the axillary nerve from its origin from the posterior cord to its bifurcation was 44.0 ± 10.5 mm on average. This length was 6.5 mm longer in males than in females, which was a statistically significant difference (p < 0.05).

Ramification of the muscular branches from the anterior division was distal to that of the muscular branches from the posterior division. The location of the first ramification of a muscular branch from the anterior division was an average of 23.6 ± 10.0 mm from the bifurcation. The first ramification of a muscular branch from the posterior division was an average of 16.9 ± 9.4 mm from the bifurcation. The first ramification of a muscular branch from the anterior division was distal to the quadrangular space in 78.0% of upper limbs. In contrast, the first ramification of a muscular branch from the posterior division was proximal to the quadrangular space in 66.0% of upper limbs.

In 82.0% of upper limbs, the diameter of the anterior division was larger than the diameter of the posterior division. The average diameters of the axillary nerve, the anterior division, and the posterior division were 3.0 ± 0.5 mm, 2.4 ± 0.5 mm, and 2.0 ± 0.5 mm, respectively.

### Divisions and muscular branches of the axillary nerve

The average number of deltoid branches from the axillary nerve was 11.1 ± 2.4. The spinous part of the deltoid muscle was innervated by the smallest number of nerve branches among the parts (Table 1). Mixed type axillary nerves provided more branches to the spinous part than the other axillary nerve types (p < 0.05). The number of branches to the spinous part was 2.5 for mixed type axillary nerves, and 57.7% of these were from the anterior division.

**Table 1:** Number and lengths of each part of the axillary nerve.

	Spinous part	Acromial part	Clavicular part
Number of branches	2.0 ± 0.9 (1–4)	4.9 ± 2.1 (2–9)	4.2 ± 1.5 (1–8)
Length	46.3 ± 14.7	22.4 ± 11.9	9.4 ± 3.7

*p* < 0.05.

The average lengths of the terminal branches from the main division were 9.4 ± 3.7 mm to the clavicular part, 22.4 ± 11.9 mm to the acromial part, and 46.3 ± 14.7 mm to the spinous part. Branch lengths to the spinous part were greater on the left than the right with statistical significance (p < 0.05).

### Site of junction of the nerve branch to the deltoid muscle

**Figure 3:**
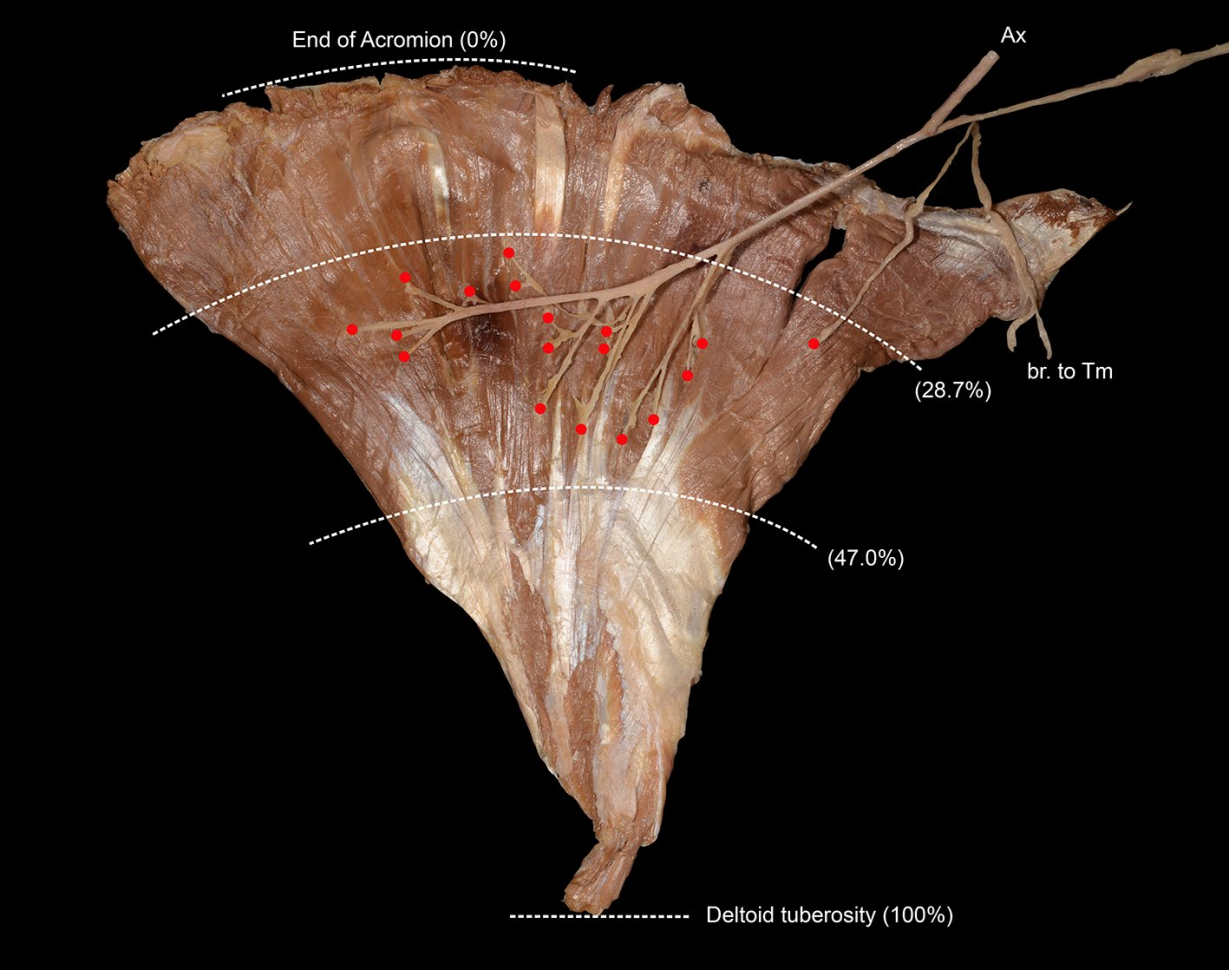
Entry points of axillary nerve branches to the deltoid muscle. Nerve terminals onto the deltoid muscle are concentrated between the upper quarter and middle of the muscle. *Tm* teres minor muscle.

Nerve terminals on the deltoid muscle were concentrated in the second upper quarter of the deltoid muscle. The junction of nerve branches to the deltoid muscle was located between the level of 28.7 ± 7.6% and the level of 47.0 ± 6.7% from the acromial tip to the deltoid tuberosity (Fig. 3). In 80.0% of shoulders, the highest entry point of nerve branches into the deltoid muscle was the acromial part. The lowest entry point was also in the acromial part in 86.0% of shoulders, with a diamond-shaped area of entry.

### Teres minor branch of the axillary nerve

In all upper limbs, the teres minor muscle was innervated only by the posterior division of the axillary nerve. The mean number of branches innervating the teres minor was 1.5 ± 0.6, and the mean length of the longest branch to the teres minor was 36.8 ± 11.2 mm. Entry points of nerve branches were on the deeper side of the teres minor muscle.

### Anatomy of the radial nerve to the triceps brachii muscle

All muscular branches to the triceps brachii muscle branched from the radial nerve before passing through a triangular inlet. Each head of the triceps brachii muscle was innervated by branches that arose separately from the radial nerve or by common branches for multiple triceps heads. In 74.0% of upper limbs, there was a common branch to the medial and lateral heads. In contrast, in 26.0% of upper limbs, all triceps heads were innervated by muscular nervous branches that arose from the radial nerve separately.

There were more branches to the long head than to the other heads. The average numbers of branches to the long head, lateral head, medial head, and common branch to the lateral and medial heads were 2.1 ± 0.8, 1.6 ± 0.9, 1.5 ± 1.0, and 0.7 ± 0.6, respectively. The number of branches was greater for the long and lateral heads than for the medial head, with statistical significance (p < 0.05).

The nerve branch to the medial head was longer than the branches to the other heads. Lengths of the medial head branch, lateral head branch, long head branch, and the common branch to the lateral and medial heads were 63.5 ± 36.4 mm, 38.1 ± 26.7 mm, 23.2 ± 19.5 mm, and 48.5 ± 25.8 mm, respectively.

Branches to the long head had a larger diameter than branches to the other heads. Diameters of branches to the long head, lateral head, and medial head were 1.4 ± 0.3 mm, 1.3 ± 0.3 mm, and 1.1 ± 0.4 mm, respectively. The average diameter of common branches to the lateral and medial heads was 1.7 ± 0.4 mm, which was significantly larger than that of branches to each head.

### Cross-section between the axillary nerve and radial nerve

**Figure 4:**
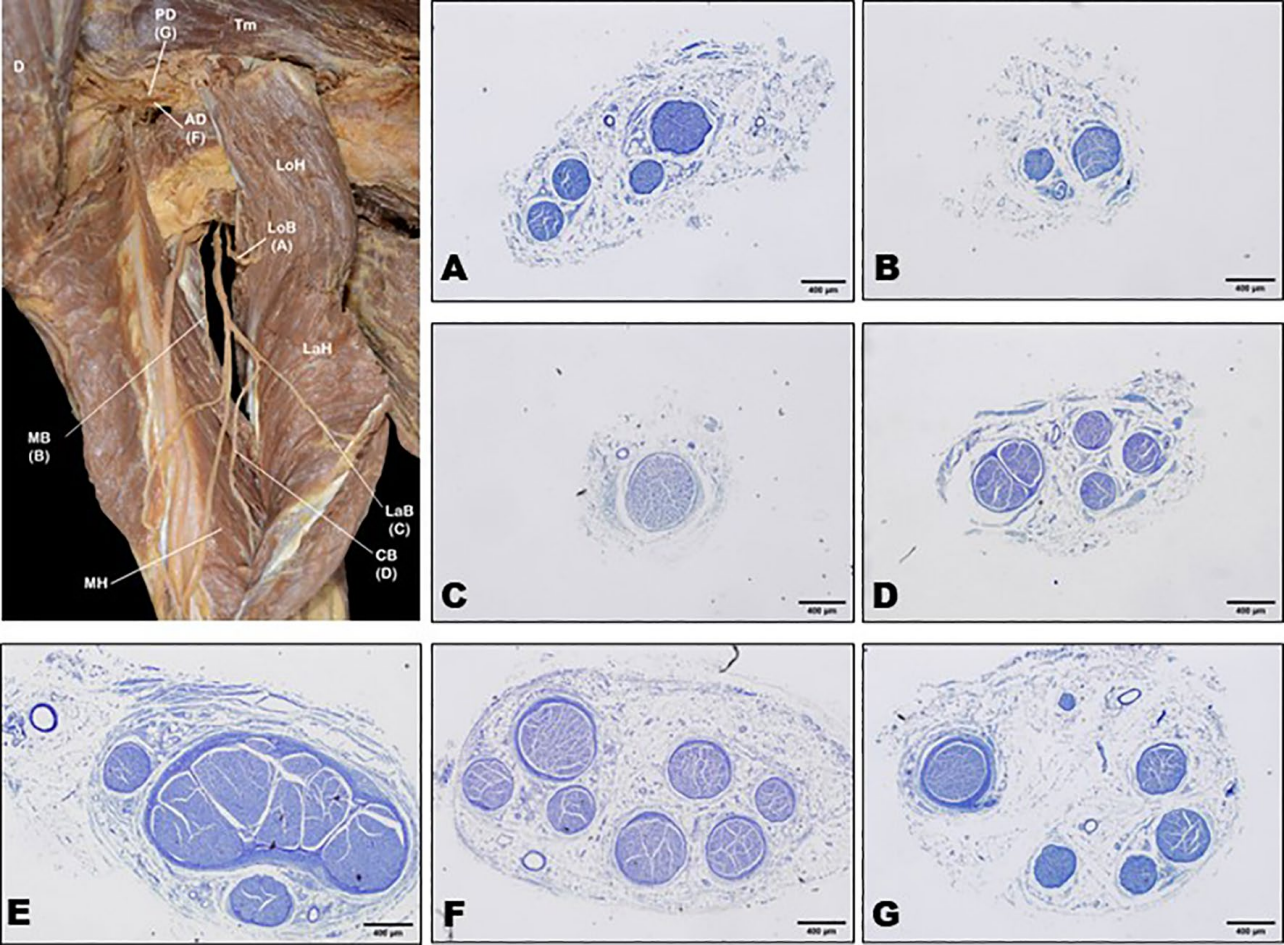
Cross-sections of the axillary nerve and radial nerve. Obtained tissue sections were serially sectioned into 2-μm-thick slices and stained with Toluidine blue O. (**A**) Long branch of the radial nerve, (**B**) medial branch of the radial nerve, (**C**) lateral branch of the radial nerve, (**D**) common branch of the medial and lateral branch of the radial nerve, (**E**) axillary nerve, (**F**) anterior division of the axillary nerve, and (**G**) posterior division of the axillary nerve. *AD* anterior division of the axillary nerve, *PD* posterior division of the axillary nerve, *CB* common branch of the medial and lateral branch of the radial nerve, *LaB* lateral branch of the radial nerve, *LoB* long branch of the radial nerve, *MB* medial branch of the radial nerve, *D* deltoid muscle, *LoH* long head of the triceps brachii muscle, *MH* medial head of the triceps brachii muscle.

To identify the most suitable branch for nerve transfer, the diameter and axon numbers per nit area of branches between the axillary and radial nerves were compared. The diameter of the muscular branch was larger in the axillary nerve and its divisions than in the radial nerve and the branches to the triceps brachii (Table 2). The mean diameter of the axillary nerve before division was 3.0 ± 0.5 mm. In the anterior and posterior divisions, this diameter was 2.5 ± 0.6 mm and 2.3 ± 0.6 mm, respectively. In the radial nerve, the diameters of branches to the triceps brachii long head, lateral head, medial head, and common branch of the medial and lateral head were 1.4 ± 0.3 mm, 1.3 ± 0.3 mm, 1.1 ± 0.4 mm, and 1.7 ± 0.4 mm, respectively. In cross-section, the mean number of axons was 214.8 ± 43 and the number of axons in a 0.04 mm^2^ area was similar among all branches (Fig. 4).

**Table 2:** Diameter and axon number within axillary and radial nerve fascicles.

	Diameter	Axon number per unit area
Axillary nerve	3.0 ± 0.5 (2.36–3.96)	218.0 ± 28.6 (182–262)
Anterior division	2.5 ± 0.6 (1.23–3.08)	225.3 ± 31.0 (195–294)
Posterior division	2.3 ± 0.6 (1.70–3.55)	206.4 ± 37.6 (136–271)
Radial nerve		
Long branch	1.4 ± 0.3 (1.08–1.79)	222.1 ± 67.3 (164–352)
Lateral branch	1.3 ± 0.3 (0.53–1.78)	231.3 ± 61.7 (154–379)
Medial branch	1.1 ± 0.4 (0.92–1.81)	196.7 ± 45.7 (135–256)
Common branch	1.7 ± 0.4 (1.18–2.33)	207.3 ± 18.9 (169–238)

Data presented as mean ± SD, unit area: 0.04 mm^2^.

*p* < 0.05.

## Discussion

The axillary nerve ran a mean distance of 44.0 mm in the axilla and divided into anterior and posterior divisions before passing through the quadrangular space in every specimen we examined. Early bifurcation of the axillary nerve before passing through the quadrangular space is an expected finding because of innervation of the teres minor muscle on the muscle’s deep surface, which is proximal to the quadrangular space. If the division was distal to the space, branches to the teres minor muscle would have to take a recurrent pathway to re-enter the axilla from the posterior side of the arm. The axillary nerve is one of the most commonly injured nerves during surgical procedures of the shoulder^4,11–13^ and axillary nerve injuries account for 6–10% of all brachial plexus injuries^11,12^.The entire axillary nerve or its divisions can be damaged by an injury near the quadrangular space. The axillary nerve is damaged most commonly at the posterior opening of the quadrangular space^3^. Owing to its early bifurcation, when the axillary nerve is injured near the quadrangular space, innervation of the teres minor muscle is protected by the muscle itself, as there is no superficial exposure to the quadrangular space. In contrast, denervation of the deltoid muscle can be caused by injury of the anterior division or injury of single muscular branches^13–15^. Each division of the axillary nerve is related to different movements of the glenohumeral joint. Therefore, the function of individual branches in terms of their effect on the deltoid muscle should be considered when performing nerve replacement. In shoulders with a mixed type axillary nerve where the spinous part of the deltoid is innervated by both divisions, the anterior division is responsible for medial rotation, flexion, abduction, extension, and lateral rotation of the shoulder; while the posterior division is responsible for extension and lateral rotation of the shoulder by the deltoid and teres minor muscles. In the anterior dominant type, the anterior division contributes to medial rotation, flexion, abduction, extension, and lateral rotation of the shoulder; while the posterior division is responsible only for extension and lateral rotation of the shoulder by the teres minor muscle. In the posterior dominant type, the anterior division helps the shoulder rotate medially, flex, and abduct but is not involved in lateral rotation or extension of the glenohumeral joint, which is performed by only the posterior division. This type corresponds with the nerve distribution of the deltoid muscle as described by Frohse and Frenkel^16^. In these types, the anterior division is important for abduction of the shoulder, for which other muscles such as the supraspinatus cannot compensate properly. In contrast, dysfunction of the teres minor and spinous part of the deltoid can be compensated by other muscles such as the infraspinatus, teres major, and latissimus dorsi^17–19^. Therefore, the posterior division may not be as important as the anterior division. Damage of the anterior division is therefore more serious than damage of the posterior division because of the possibility of compensation by other muscles in the latter case. The axillary nerve is vulnerable to injury from intramuscular injection of the deltoid muscle for vaccination^20^. In the present study, junctions of the axillary nerve branches to the deltoid muscle were confined to the second upper quarter of the muscle (Fig. 3). In most cases, the thickness of the deltoid muscle will protect against needle penetration^21^. However, especially in thin patients with small deltoid muscles, avoidance of this area during needle insertion is recommended. The distribution of the junctions is also important to consider when performing intraosseous infusion into the humeral head. During intraosseous access, the humerus is medially rotated, and the needle is inserted into the greater tubercle through its superolateral surface at a 45° angle to the horizontal plane^22^. According to our findings, insertion of the intraosseous needle is superior to the area of nerve-muscle junctions and favorable. There are two important principles to maximize the outcomes of nerve transfers. The first is to reinnervate the recipient nerve as close to the target muscle as possible^23^. Although some studies recommend nerve transfer using the branch to the long head of triceps brachii using a posterior approach^10,24^, the length of this branch was relatively short in our study. Because of the possibility of damage to the posterior branch of the axillary nerve and difficulty in dissecting the teres minor branch through a posterior approach^25,26^, a deltopectoral approach (anterior approach) is preferable for axillary nerve transfer ^7,26^. The radial nerve branch to the lateral head is not easily accessible because it is covered by the triceps brachii muscle as it usually arises from the radial groove. Instead, use of the branch to the medial head of triceps brachii is recommended for restoration of the deltoid muscle. The medial branch of the radial nerve is easy to find because it runs across the medial head superficially. The second principle is to use combinations of similarly behaving neuromuscular units, maximized when agonistic donor and recipients are chosen, as cortical readaptation is the physiological basis for functional recovery^23^. In our study, the number of axons in a given cross-sectional area was similar between nerve branches, which means that the number of axons is proportional to the diameter of the nerve branch. It is reasonable to transfer a donor nerve with a similar diameter to the recipient nerve.

This study has several limitations. The most significant limitation is that the measurement of lengths was conducted on formalin-fixed cadavers, which may result in deviations due to tissue shrinkage and damage from microdissection, potentially causing differences from values measured in living subjects. Additionally, the limited number of samples used for tissue staining means the findings may not fully represent the variation seen in a larger population, indicating the need for further studies with increased sample sizes to validate and extend our conclusions.

## Conclusions

The significant role of the axillary nerve in maintaining deltoid muscle functionality and in preventing palsy or malfunction highlights its vital importance in clinical and surgical procedures. Functional loss of the anterior division may be more critical because it can lead to difficulty in abduction by the acromial part of the deltoid^5^. Consequently, for axillary nerve transfer, the medial and long branches of the radial nerve, with their suitable length and axon density, are recommended as optimal donor choices for effective functional restoration.

## Materials and methods

Fifty upper extremities, 24 right and 26 left, from 29 formalin-embalmed cadavers (mean age, 75.8 years; range 52–95 years) with no pathologies or surgical history in the upper extremity were used in this study. All study procedures approved by the Surgical Anatomy Education Centre, Yonsei University College of Medicine (approval number: YSAEC: 23-006). The participants have provided informed consent to donate their bodies for research purposes. The authors state that every effort was made to follow all local and international ethical guidelines and law that pertain to the use of human cadaveric donors in anatomical research^27^.

After removal of the skin and subcutaneous tissue of the shoulder, the deltoid and teres minor muscles were identified. The deltoid was detached from its origin on the clavicle, acromion, and scapular spine and divided into three parts according to origin. The axillary nerve was traced from the level of the quadrangular space to the points of entry into the perimysium of the deltoid and teres minor muscles. Number and length of axillary nerve branches from the posterior and anterior divisions were compared. We measured the location where the axillary nerve entered the deltoid muscle, with the acromial end as 0% and the deltoid tuberosity as 100%.

To expose the medial branch of the radial nerve, the lateral head of the triceps brachii was divided. Other branches of the radial nerve were traced proximally to their origins from the radial nerve. The number, diameter, and length of branches were recorded.

From ten sides of fresh cadavers, 5-mm-long segments were harvested from the main trunk of the axillary nerve, its anterior and posterior divisions, and radial branches, to estimate the number of axonal fibers in these nerve branches. Branches were fixed overnight in 4% paraformaldehyde, dehydrated, and then cleared in toluene. They were then embedded in paraffin and cut into transverse sections of 2 µm thickness. Sections were stained with toluidine blue and photographed with a microscope-mounted camera. Morphometric measurements were performed at 100-fold magnification. The number of axons in randomly selected 0.04 mm^2^ cross sectional areas was measured by ImageJ software version 1.53t (NIH, Bethesda, MD, USA).

Independent t-tests and chi-square tests were used to compare the significance of differences between body sides or genders. Statistical analyses were performed using the software SPSS version 21 (IBM SPSS Software, Armonk, NY, USA). One-way ANOVA was used to verify the significance of differences in measurements between the types of nerves.

## Data Availability

The datasets generated during and/or analysed during the current study are available from the corresponding author on reasonable request.
